# Anticholinergic delirium following *Datura stramonium* ingestion: Implications for the Internet age

**DOI:** 10.4103/0974-2700.66565

**Published:** 2010

**Authors:** David Vearrier, Michael I Greenberg

**Affiliations:** Department of Emergency Medicine, Drexel University College of Medicine, 245 N 15^th^ St, Mail Stop #1011, Philadelphia, PA, USA

**Keywords:** Cholinergic antagonists, Datura, Internet, street drugs, young adult

## Abstract

Recreational use of Datura to deliberately induce an anticholinergic delirium is not uncommon. We present a case of Datura intoxication in a young adult who learned about the recreational use of Datura on the Internet and subsequently purchased *Datura stramonium* seeds from an online vendor. Using the Google search engine, we conducted searches for “Datura,” “jimson weed” and “Datura seeds” and reviewed the first 200 search results for each search term. We found 16 websites recommending the recreational use of Datura, 12 vendors selling seeds of genus Datura and one website that both promoted the recreational use of Datura and also sold *Datura stramonium* leaves. The promotion of recreational use of Datura on the Internet represents a danger to public health and the ability to purchase Datura seeds from Internet vendors may increase the prevalence of Datura abuse.

## INTRODUCTION

Plants of the genus Datura contain tropane alkaloids with significant anticholinergic activity in humans. Today, recreational use of Datura to deliberately induce an anticholinergic delirium has been most frequently reported among adolescents and young adults.[1–3] We report a case of altered mental status in a 22-year-old man following the ingestion of vodka and seeds of *Datura stramonium*. After his acute intoxication resolved, the patient reported that he learned about the recreational use of Datura on the Internet and subsequently purchased *Datura stramonium* seeds from an online vendor.

## CASE HISTORY

**Figure 1:**
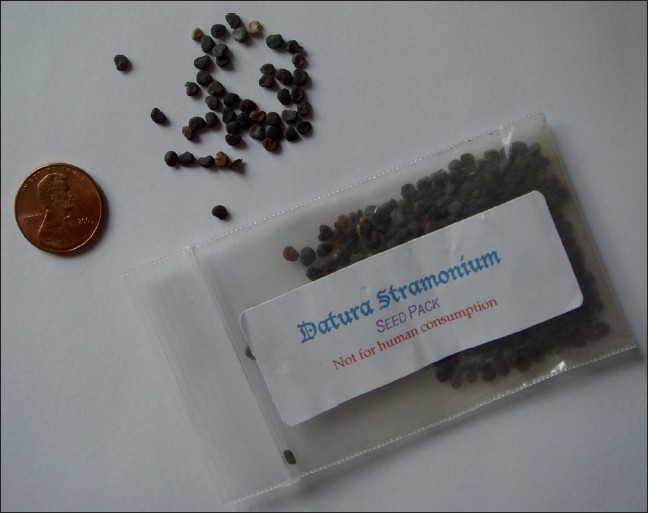
Plastic bag containing Datura seeds found in the patient’s pocket

A 22-year-old man was brought to the emergency department by paramedics for altered mental status. The patient was unable to provide any history secondary to his altered mental status, although a friend relates that the patient drank “10 glasses” of vodka and had been chewing on seeds to “get high.” A search of the patient’s pockets revealed a packet of seeds labelled “*Datura stramonium* - not for human consumption” [Figure 1].

An initial set of vital signs were remarkable only for mild tachypnea, with a respiratory rate of 26 breaths per minute. Physical exam was remarkable for a male appearing stated age with somnolence interrupted by episodes of psychomotor agitation, who was not responding appropriately to the questions being asked. His pupils were 9 mm bilaterally and sluggishly reacted to light to 8 mm. He had an odor of alcohol on his breath and partially chewed black seeds were present in the oropharynx. Pulmonary, cardiac and extremity exams were normal. Abdominal exam was significant for hypoactive bowel sounds. His skin was warm, dry and flushed. Neurological exam was limited by a lack of patient cooperation, but no gross neurological deficit was present, the patient was moving all extremities, the patient withdrew to pain in all four extremities and biceps and patellar reflexes were normal bilaterally. The Glasgow Coma Score was nine. Laboratory studies were significant only for a serum ethanol level of 330 mg/dl.

The patient was diagnosed with anticholinergic toxicity and alcohol intoxication. Two doses of physostigmine 1 mg were administered intravenously 5 min apart without significant improvement in the patient’s mental status. The patient was noted to no longer have a gag reflex and was subsequently endrotracheally intubated for airway protection.

After intubation, an orogastric tube was placed and whole bowel irrigation with polyethylene glycol solution was initiated. The remainder of the patient’s clinical course was unremarkable, with the patient extubating himself 36 h after intubation. The patient was able, at that time, to provide us with additional history as to his use of Datura. He learned about the Datura while browsing a website containing information about recreational use of a variety of licit and illicit substances. From the website, he learned about the routes of administration of Datura and the expected psychological and physiological effects. He reported that he subsequently purchased Datura seeds from an Internet vendor and that he occasionally ingests Datura seeds with alcohol “to get high.”

## DISCUSSION

Recreational use of Datura seems to be on the rise among adolescents and young adults over the last several decades.[4] While specific data on the changing rates of Datura abuse in the United States is not available, a review of the epidemiology of intentional anticholinergic plant poisonings, as reported by the American Association of Poison Control Center’s National Poison Data Base (NPDS), suggests an increasing incidence of Datura abuse. In the first 5 years of data collection (1983–1987), the NPDS reported an average of 74 intentional anticholinergic plant poisonings annually, while in the most recent 5 years (2004–2008), 427 such poisonings occurred annually. Additionally, the percent of all anticholinergic plant poisonings that were due to intentional exposure increased from 22% to 44% over the same period.[5]

It has been reported that information on psychoactive substances on the Internet influences drug-use behaviors in adolescents.[6] Some authors believe that the Internet may be influencing the epidemiology of Datura use.[7] The Internet allows the dissemination of information on Datura to adolescents and young adults who otherwise might not have learned of its psychotropic properties. Websites offer information on intended effects following Datura use, side-effects, methods of use and share experiences.[8–10] In some cases, quite detailed information is available on how to prepare Datura in cigarettes, teas and other preparations.[11] While some websites may also discuss adverse-effects, others minimize or completely omit this information, in effect, promoting recreational use of substances that may cause significant harm or death.

Historically, procuring Datura involved either foraging for the plant or purchasing seeds from a gardening store that sells Datura. However, the Internet offers easy procurement of seeds to persons wishing to recreationally use Datura. Numerous vendors sell packets of seeds of various Datura cultivars. Some vendors specifically focus on the sale of seeds, plant parts (e.g., foliage) and/or extracts of plants with abuse potential.[12] Although these sites may state warnings that the seeds are not for human consumption, this advice is easily ignored. For persons who either do not wish to forage for Datura or cannot purchase those seeds at local gardening stores, the ability to purchase such seeds from Internet vendors makes it substantially easier to recreationally use Datura.

We informally investigated the availability of information on both the recreational use of Datura and Datura seeds for sale on the Internet. Using the Google search engine (www.google.com), we conducted searches for “Datura,” “jimson weed” and “Datura seeds” and reviewed the first 200 search results for each search term. The reviewed results included 16 websites with unique domain names recommending the recreational use of Datura, with various recommendations on the administration and descriptions of psychotropic effects. The results also included 12 vendors with unique domain names selling seeds of the genus Datura. One website both promoted the recreational use of Datura and also sold *Datura stramonium* leaves. An additional 176 listings for Datura seeds were found at the online vendor, www.ebay.com, and six listings at the online vendor, www.amazon.com.

## CONCLUSION

The increasing availability of information about Datura on the Internet and the way in which it is presented represents a developing public health problem. Sale of Datura seeds by Internet vendors may make it substantially easier to recreationally use Datura, potentially increasing the prevalence of Datura abuse. Public health efforts should be initiated to educate adolescents and young adults, parents and school health managers about this potentially lethal substance of abuse. Clinicians should maintain a high level of suspicion for Datura abuse in young patients with unexplained anticholinergic toxicity.
